# Imaging features in post-mortem x-ray dark-field chest radiographs and correlation with conventional x-ray and CT

**DOI:** 10.1186/s41747-019-0104-7

**Published:** 2019-07-11

**Authors:** Alexander A. Fingerle, Fabio De Marco, Jana Andrejewski, Konstantin Willer, Lukas B. Gromann, Wolfgang Noichl, Fabian Kriner, Florian Fischer, Christian Braun, Hanns-Ingo Maack, Thomas Pralow, Thomas Koehler, Peter B. Noël, Felix Meurer, Dominik Deniffel, Andreas P. Sauter, Bernhard Haller, Daniela Pfeiffer, Ernst J. Rummeny, Julia Herzen, Franz Pfeiffer

**Affiliations:** 1 Department of Diagnostic and Interventional Radiology, School of Medicine & Klinikum rechts der Isar, Technical University of Munich, 81675 Munich, Germany; 2 Chair of Biomedical Physics, Department of Physics and Munich School of BioEngineering, Technical University of Munich, 85748 Garching, Germany; 3 Institute of Forensic Medicine, Ludwig-Maximilians-University Munich, 80336 Munich, Germany; 4 Philips Medical Systems DMC GmbH, 22335 Hamburg, Germany; 5 Philips GmbH Innovative Technologies, Research Laboratories, 22335 Hamburg, Germany; 6 Institute for Advanced Study, Technical University of Munich, 85748 Garching, Germany; 7 Institute of Medical Informatics, Statistics and Epidemiology, Technical University of Munich, 81675 Munich, Germany

**Keywords:** Lung, Observer variation, Radiography (thoracic), Tomography (x-ray computed), X-ray dark-field imaging

## Abstract

**Background:**

Although x-ray dark-field imaging has been intensively investigated for lung imaging in different animal models, there is very limited data about imaging features in the human lungs. Therefore, in this work, a reader study on nine post-mortem human chest x-ray dark-field radiographs was performed to evaluate dark-field signal strength in the lungs, intraobserver and interobserver agreement, and image quality and to correlate with findings of conventional x-ray and CT.

**Methods:**

In this prospective work, chest x-ray dark-field radiography with a tube voltage of 70 kVp was performed post-mortem on nine humans (3 females, 6 males, age range 52–88 years). Visual quantification of dark-field and transmission signals in the lungs was performed by three radiologists. Results were compared to findings on conventional x-rays and 256-slice computed tomography. Image quality was evaluated. For ordinal data, median, range, and dot plots with medians and 95% confidence intervals are presented; intraobserver and interobserver agreement were determined using weighted Cohen *κ*.

**Results:**

Dark-field signal grading showed significant differences between upper and middle (*p* = 0.004–0.016, readers 1–3) as well as upper and lower zones (*p* = 0.004–0.016, readers 1–2). Median transmission grading was indifferent between all lung regions. Intraobserver and interobserver agreements were substantial to almost perfect for grading of both dark-field (*κ* = 0.793–0.971 and *κ* = 0.828–0.893) and transmission images (*κ* = 0.790–0.918 and *κ* = 0.700–0.772). Pulmonary infiltrates correlated with areas of reduced dark-field signal. Image quality was rated good for dark-field images.

**Conclusions:**

Chest x-ray dark-field images provide information of the lungs complementary to conventional x-ray and allow reliable visual quantification of dark-field signal strength.

**Electronic supplementary material:**

The online version of this article (10.1186/s41747-019-0104-7) contains supplementary material, which is available to authorized users.

## Key points

- X-ray dark-field chest radiographs provide information complementary to conventional chest x-ray
- Dark-field signal shows apicobasal gradient in the human lungs
- Dark-field signal can be reliably quantified by visual assessment
- Pulmonary infiltrates, cardiomegaly, and haemopericardium can reduce dark-field signal

## Background

The discovery of *x* radiation marks the birth of diagnostic radiology and its use remains indispensable in daily clinical practice. However, even modern computed tomography (CT) imaging exploits only part of the physical interactions between x-rays and matter for contrast formation in x-ray-based images. Similar to visible light, x-rays can not only be interpreted as particles, but also show wave-like properties, such as refraction, that can be utilised for contrast formation [1, 2]. A grating-based approach has been intensively investigated for its application in biomedical imaging [3, 4]. Grating-based x-ray dark-field imaging allows detection, quantification, and visualisation of small-angle x-ray scattering [5], which is not possible with conventional x-ray imaging devices. This technique has been translated to the use of conventional x-ray sources [6].

Small-angle x-ray scattering occurs at interfaces between structures of different electron density, *e.g.*, air-tissue interfaces in the lungs or bone-fat interfaces in the spongious bone. In the dark-field x-ray image, the strength of the dark-field signal represents the amount of small-angle x-ray scattering. Due to its specific histologic anatomy, with numerous air-tissue interfaces at the microscopic level of the alveoli, the lungs are of special interest for x-ray dark-field imaging. In 2013, it was already shown in a mouse model that normal lungs generate a high signal on x-ray dark-field radiography [7]. Further small animal studies have demonstrated the capability of x-ray dark-field imaging to detect and quantify pulmonary emphysema with significantly higher sensitivities compared to conventional radiography [8–12].

In animal studies, x-ray dark-field radiography has also shown better diagnostic performance than conventional x-ray for the detection of pulmonary fibrosis [13, 14], lung cancer [15, 16], pneumothorax [17, 18], neonatal lung injury [19], and acute lung inflammation [20]. As early imaging setups were optimised for small animals, the technology was further developed to enable imaging of human-sized animals. This has been demonstrated in a large animal model [18, 21] and, finally, in the first x-ray dark-field chest radiograph of a human body [22].

So far, however, imaging features of x-ray dark-field radiography have not been described from the perspective of clinical radiology. To allow x-ray dark-field imaging to become a clinical imaging modality, imaging findings have to be reported in a consistent manner and correlated with established imaging modalities to facilitate correct interpretation.

Therefore, the purpose of this study on post-mortem human chest x-ray dark-field radiographs was to address dark-field signal strength in the lungs, inter- and intraobserver agreement, and image quality and to correlate findings with conventional x-ray and CT.

## Methods

### Human bodies

This prospective study was approved by the Institutional Review Board and was conducted between November 2015 and July 2018. Human bodies were transferred to the Institute of Forensic Medicine at coroner’s inquest. Due to the mode of inclusion, no preselection of the human bodies according to certain criteria, *e.g.*, the presence of specific lung diseases, was possible. Externally visible conditions causing a significant impairment of the normal thoracic anatomy and signs of advanced decomposition were exclusion criteria. Imaging was performed before autopsy no longer than 36 h after death with bodies cooled to slow decomposition. The imaging was not part of the forensic analysis. Altogether, nine bodies (3 females, age range 52–88 years; 6 males, age range 60–83 years) were imaged. Airway pressure was kept constant (20–25 mbar) during x-ray dark-field imaging by endotracheal intubation and mechanical ventilation.

### X-ray dark-field imaging

The setup was previously described [18, 21]. The employed three-grating arrangement is asymmetric [periodicity of G0, G1, and G2 is 68.72 μm, 8.73 μm, and 10 μm, respectively; inter-grating distances: *d*(G0–G1) = 1.60 m, *d*(G1–G2) = 0.25 m]. Gold heights for all gratings range between 150 and 200 μm. As already described [23], the shadow of G1 is directly projected onto G2. G1 and G2 are tiled to each cover an area of 40 × 2.5 cm^2^. The tiling procedure has been already described [24]. All gratings are mounted on a swing pivoting around the focal spot. Acquisition is performed via fringe-scanning, yielding a field of view of 32 × 35 cm^2^. The source (MRC 200 0310 ROT-GS 1004, Philips Medical Systems, Hamburg, Germany) is an actively cooled tungsten rotating anode and was operated at 70 kVp, where a mean visibility of 31% was achieved. A flat panel detector (Pixium RF 4343, Trixell, Moirans, France) was used. Source and detector remain stationary during acquisition. Imaging was performed in supine position with anterior-posterior beam setup. Acquisition time was 40 s. For additional information, see Additional file 1: Figure S1.

### CT imaging

Human bodies were imaged in supine position on a 256-slice CT unit (Brilliance iCT, Philips, Amsterdam, Netherlands). High-resolution chest CT was performed in craniocaudal direction with 128 × 0.625 mm collimation and 0.383 pitch. Tube voltage was 120 kVp. Mean tube current was 537 mA. CT images were reconstructed with iDose^4^, a hybrid iterative reconstruction technique (Philips, Amsterdam, Netherlands), at level 2 in axial, coronal, and sagittal view with a slice thickness of 3 mm, 1024 × 1024 matrix, and 350-mm field of view.

### Data acquisition and processing of x-ray dark-field imaging

The fringe-scanning method [25] was used for data acquisition: a fringe pattern is induced on the detector by detuning inter-grating distances. Acquisition while moving the pattern across the sample produces images of the same features at multiple relative grating shifts.

Signal extraction was performed using the least-squares minimisation of an image formation model similar to the one presented in [26]. To correct for visibility reduction due to beam-hardening, a correction algorithm comparable to the method presented in [27] was used.

The dark-field radiographs were low-pass-filtered (2D Gaussian filter kernel, *σ* = 3.2 pixels). This reduces noise levels by a factor of ~ 11.3 for white noise, leading to a visual impression more similar to conventional radiography. Although low-pass filtering obscures small features, these are nearly undetectable in the unfiltered images due to the high noise levels. The used kernel size was found to be an acceptable trade-off between image impression and resolution. No filtering was applied to conventional radiographs.

### Reader study

Visual image analysis was independently performed by three residents with 3 (F. M.), 5 (A. S.), and 5 (D. D.) years of experience in chest imaging on a clinical Picture Archiving and Communication System workstation. For training purposes, the dark-field radiograph of human body 4 was presented before the reading session to demonstrate low and high dark-field signal intensity. In the first reading session, window settings were fixed to allow optimal comparison of low and high dark-field signal intensities in dark-field radiographs and opacification in conventional chest x-rays to avoid influence of individual windowing. A linear mapping between grey values and logarithmic visibility reduction ratios, − ln(*V*/*V*_0_), was used. Window level and width were set to 8,500 and 5,000, respectively. Converting these numbers back to physical quantities, this means that a logarithmic visibility reduction ratio of -0.268 corresponds to “black,” and a value of 0.343 corresponds to “white.”

**Fig. 1:**
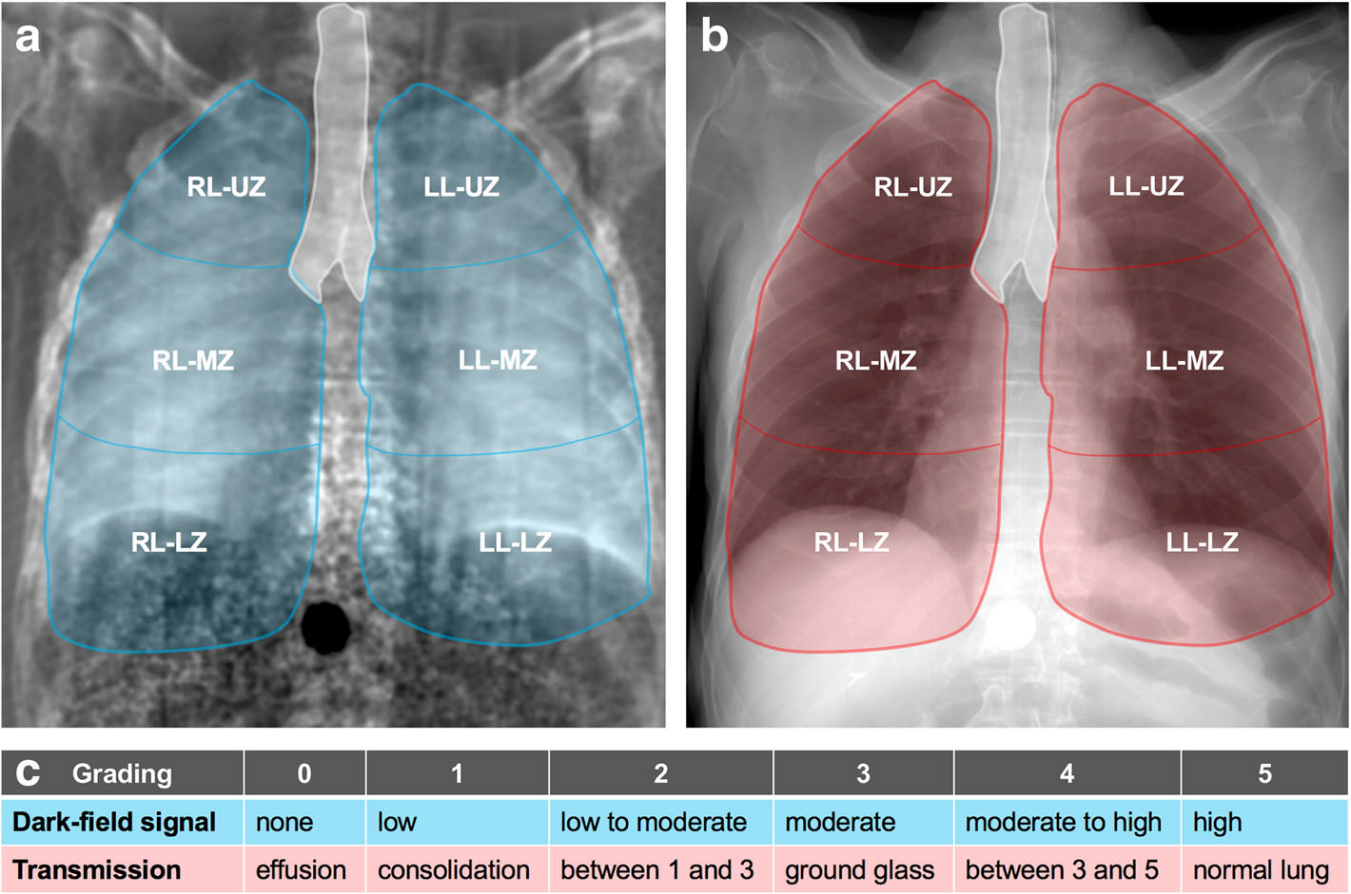
Visual evaluation scheme and grading scale for dark-field chest radiographs and conventional x-rays. In dark-field (**a**) and conventional (**b**) chest x-ray, lungs are divided into six regions: right lung-upper zone (RL-UZ), right lung-middle zone (RL-MZ), right lung-lower zone (RL-LZ), left lung-upper zone (LL-UZ), left lung-middle zone (LL-MZ), and left lung-lower zone (LL-LZ). In each region, dark-field (**a**) and transmission (**b**) signals are visually graded using a (**c**) 6-point ordinate scale

The nine x-ray dark-field radiographs had to be graded separately one after the other without the possibility to compare or change gradings. Next, the conventional x-rays were presented. On each image, the left and right lung were divided into three regions of equal height, upper, middle, and lower zones, using the apex and the costodiaphragmatic recess as anatomical landmarks. Dark-field signal intensity and degree of transmission (or opacification) of the upper, middle, and lower zones of the left and right lung were graded on a 6-point (0–5) ordinal scale (Fig. 1). For the dark-field signal intensity grading, “0” represents no (dark area in the radiograph) and “5” a high (bright area in the radiograph) dark-field signal. “1–4” represent intermediate dark-field signal intensities (Fig. 4 for comparison). For the transmission grading, “0” represents no transmission or hyperattenuation like in the clinical case of a pleural effusion where no ventilated lung parenchyma is visible. “5” represents a normal, healthy lung with high transmission or hypoattenuation. “1–4” represents intermediate transmission grades (Fig. 7 for comparison).

The reading session was repeated after 4 weeks.

In a separate reading session, the readers independently graded image quality for right and left lung on a 6-point ordinate scale: 1 = not diagnostic, 2 = sufficient, 3 = satisfactory, 4 = good, 5 = very good, and 6 = excellent. As standardised image quality criteria for dark-field radiographs do not exist, the readers were instructed to evaluate the following aspects: symmetrical reproduction of the thorax, reproduction of the whole lung, and presence of artefacts interfering with the grading of pulmonary dark-field signal intensity (*e.g.*, vertical streaking artefacts, dark-field signal from bony structures). For transmission images, the “European guidelines on quality criteria for diagnostic radiographic images” [28] were applied wherever possible considering imaging of a human body in supine position. In this setting, readers were free to change window/level values to optimise individual image impression.

### Correlation of dark-field and transmission radiography with CT findings

As there exists no data on x-ray dark-field imaging features of human lung pathologies, we performed a CT scan of each human body to correlate findings in chest CT images with signal changes in dark-field and transmission radiographs. CT images were reviewed by an attending radiologist with 10 years of experience in chest radiology (A. A. F.) using axial, sagittal, and coronal reconstructions. Pulmonary findings and extrapulmonary findings with a potential effect on dark-field signal intensity were recorded. Apart from septal thickening, the extent of pulmonary findings was visually quantified for every lobe in 10% intervals. For pleural effusions, the maximum width in anterior-posterior direction was measured in centimeters. Other findings were qualitatively recorded. CT findings were correlated with the visual assessment of dark-field signal strength in a descriptive model.

### Statistical analysis

Statistical analysis was performed using GraphPad Prism 7 for Mac OS X (Version 7.0d, GraphPad Software Inc., USA) and R version 3.4.4 (R Foundation for Statistical Computing, Vienna, Austria). For ordinal data, median and range are presented and dot plots with medians and 95% confidence interval (based on the Hodges-Lehmann method) are shown. Intraobserver and interobserver agreement of dark-field signal and transmission grading were evaluated using weighted Cohen *κ* with squared weights. Cohen *κ* coefficients with values < 0 were regarded as poor, 0–0.20 as slight, 0.21–0.40 as fair, 0.41–0.60 as moderate, 0.61–0.80 as substantial, and 0.81–1.00 as (almost) perfect agreement, according to Landis et al. [29]. Differences in distributions of dark-field signal and transmission grading for the upper, middle, and lower zones were tested separately for the right and left lung and each reader using the Friedman test. If Friedman test indicated a significant (*p* < 0.050) association between region and dark-field signal, Wilcoxon matched-pairs signed-rank test was performed for pairwise comparisons of regions of each lung for each reader. The correlation of dark-field signal with transmission grading was tested with Spearman’s rank correlation coefficient for each region of lungs and each reader. Differences in the grading of image quality between the left and right lung for dark-field and transmission radiographs, respectively, and between dark-field and transmission radiographs for left and right lung, respectively, were tested with Wilcoxon matched-pairs signed-rank test.

## Results

### Visual grading of dark-field and transmission signals

**Fig. 2:**
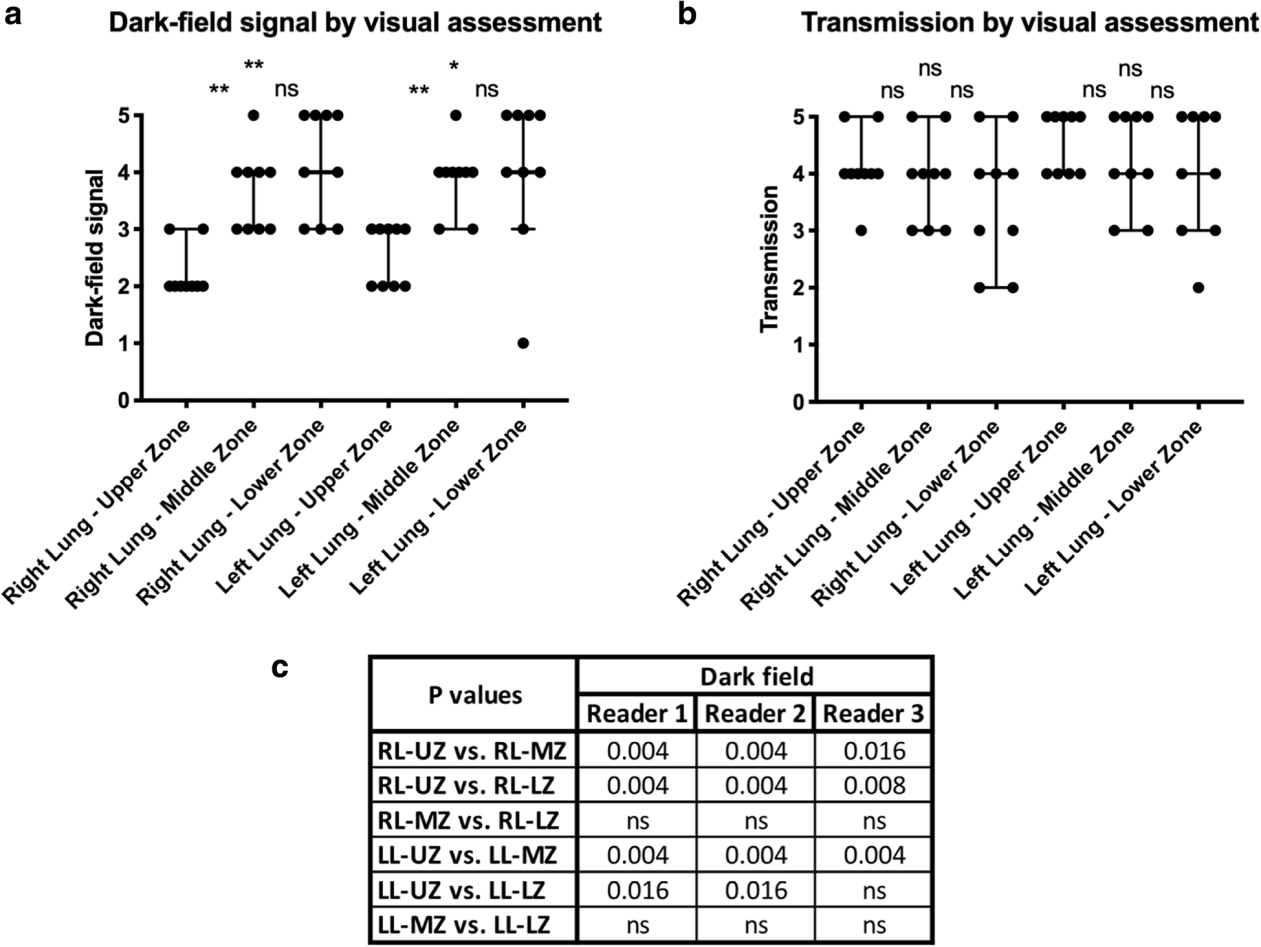
Dark-field and transmission signal grading of all nine human bodies for different regions of the lungs. Dot plot of median visual dark-field signal (**a**)/transmission (**b**) grading with 95% confidence interval on a 6-point ordinal scale (dark-field signal: 0 = no signal, 5 = maximum signal; transmission: 0 = effusion, 5 = normal lung) of all nine dark-field and transmission chest x-rays for reader 1. Dark-field signal increases from apex to base of the lung, whereas transmission shows no differences between upper, middle, and lower zones. Asterisks indicate statistical significance of median dark-field signal/transmission differences between the different zones in the left or right lobe. Associated null hypothesis probabilities in the dark-field modality for all readers are given in **c**. Non-significant differences (*p* > 0.050) are denoted by “ns”

Statistical analysis of the dark-field and transmission gradings (Table 1) indicated a significant (right lung, reader 1–3, *p* < 0.0001; left lung, reader 1, *p* = 0.001, reader 2, *p* = 0.001, reader 3, *p* = 0.001) association between the lung zones and dark-field signal, which was further investigated by pairwise comparisons of regions of each lung for each reader (Fig. 2). The median dark-field signal showed significant differences between the lung upper zones and lung middle zones. The median dark-field signal was also significantly different between the lung upper zones and lung lower zones, except for reader 3 in the left lung.

**Table 1:**
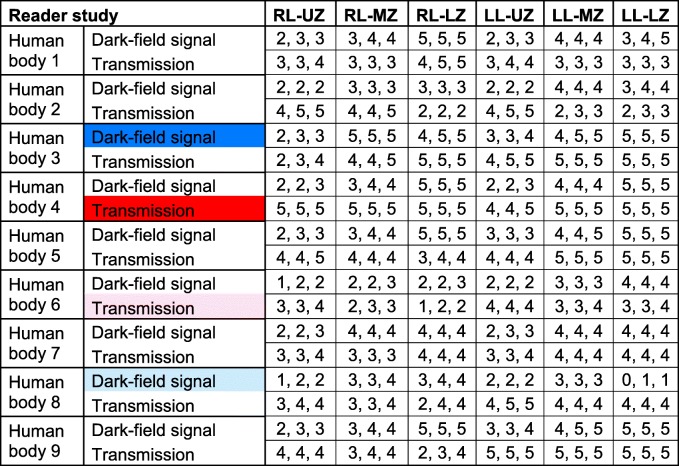
Dark-field and transmission signal grading of all nine human bodies

**Fig. 3:**
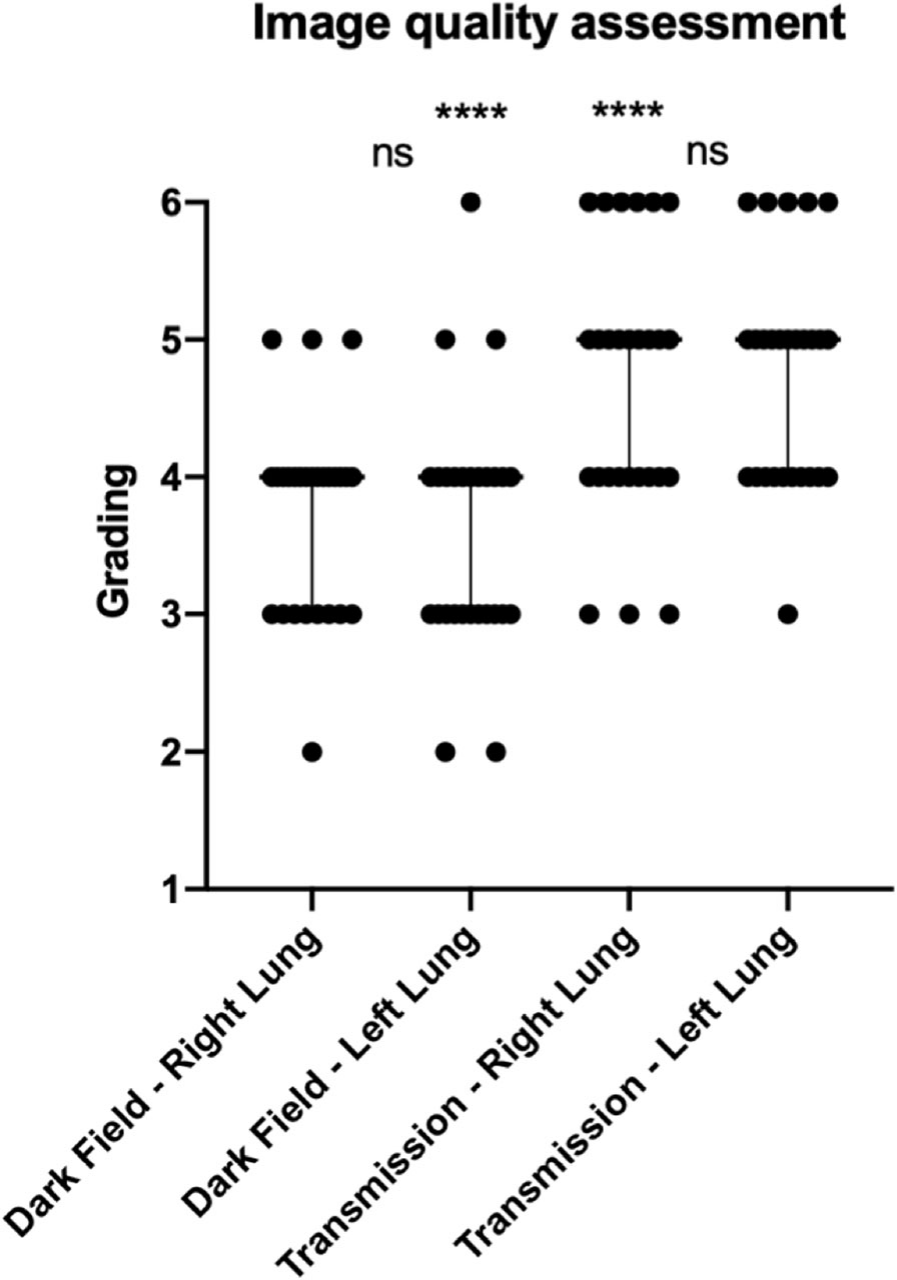
Image quality assessment of dark-field and transmission images. Dot plot of image quality grading of dark-field and transmission radiographs for the left or right lung with median and interquartile range on a 6-point ordinal scale (1 = not diagnostic, 6 = excellent) of all nine dark-field and transmission chest x-rays and all readers. Median grading is 4 and 5 for dark-field and transmission radiographs, respectively. Asterisks indicate statistical significance difference of median image quality gradings for left/right lung between dark-field and transmission radiographs. Non-significant differences (*p* > 0.050) for comparisons between left and right lung for median image quality gradings for dark-field/transmission radiographs are denoted by “ns”

**Fig. 4:**
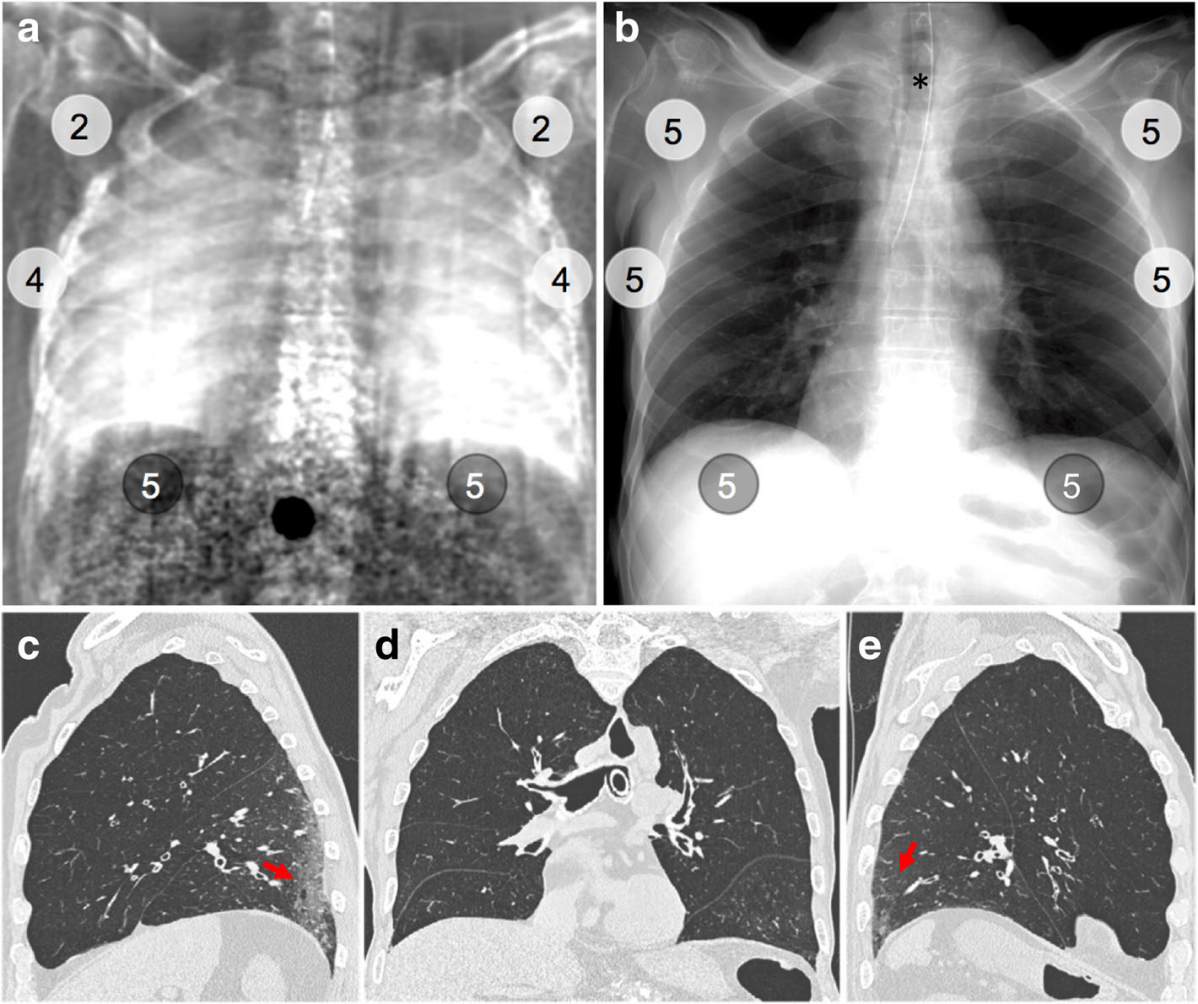
Dark-field chest radiograph, conventional x-ray, and CT of human body 4. Median visual grading (encircled numbers) of dark-field chest radiograph (**a**) by three independent readers shows increasing dark-field signal from the apex to the base of the lung. Median visual grading of transmission in conventional x-ray (**b**) shows no difference between upper, middle, and lower zones. Sagittal (**c**, **e**) and coronal (**d**) CT images show regular ventilation of emphysematous lung parenchyma except from some minor subpleural dystelectasis (red arrows) in posterior lower lobes. Asterisk marks the location of the endotracheal tube, which was repositioned between x-ray dark-field/conventional x-ray imaging and CT

**Fig. 5:**
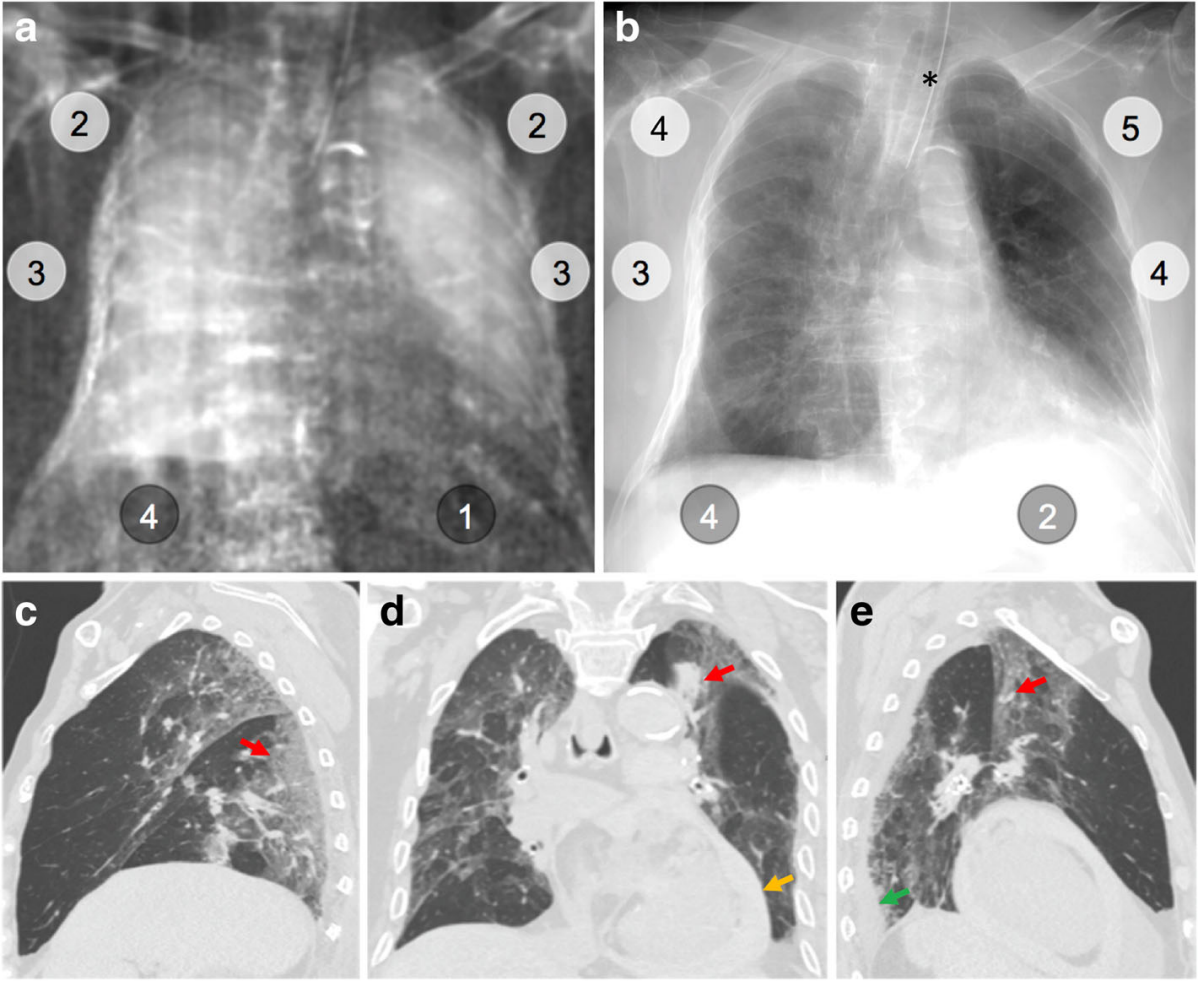
Dark-field chest radiograph, conventional x-ray, and CT of human body 8. Median visual grading (encircled numbers) of dark-field chest radiograph (**a**) by three independent readers shows a markedly reduced dark-field signal in left lung lower zone and to a minor degree in both middle zones, compared to other regions. Median visual grading of transmission in conventional x-ray (**b**) shows reduced transmission in the right lung, especially in the middle zone, and a markedly reduced transmission in the lower zone of the left lung. Sagittal (**c**, **e**) and coronal (**d**) CT images show interstitial and alveolar infiltrates (red arrows) in depending parts of the right and left upper and lower lobes. Furthermore, there is an enlargement of the heart with a significant haemopericardium (orange arrow) and small left-sided pleural effusion (green arrow). Asterisk marks the location of the endotracheal tube

**Fig. 6:**
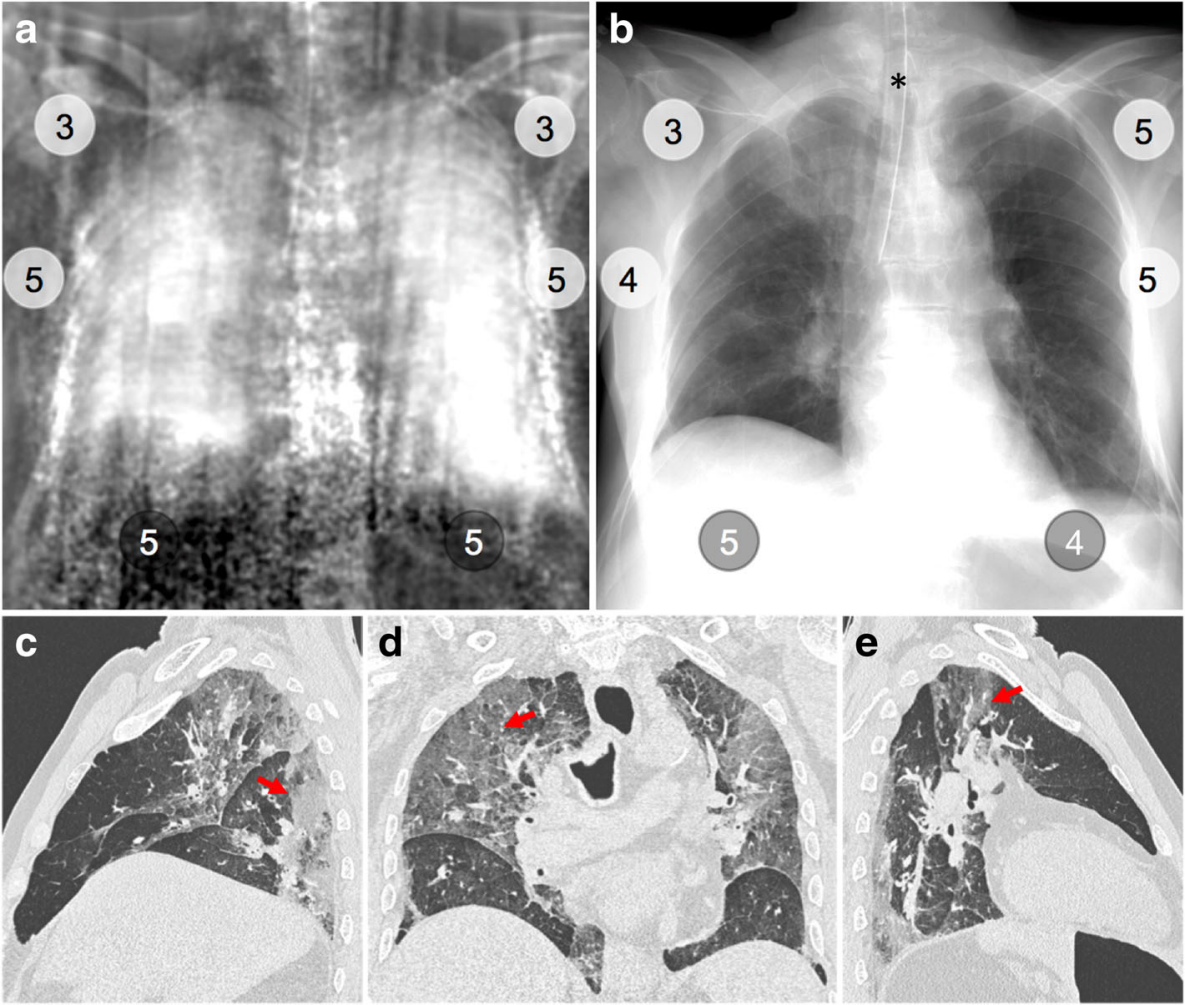
Dark-field chest radiograph, conventional x-ray, and CT of human body 3. Median visual grading (encircled numbers) of dark-field chest radiograph (**a**) by three independent readers shows highest dark-field signal in the middle and lower zones of the lung. Median visual grading of transmission in conventional x-ray (**b**) shows reduced transmission in the right lung upper zone and more pronounced in the left lung lower zone. Sagittal (**c**, **e**) and coronal (**d**) CT images show interstitial and alveolar infiltrates (red arrows) in dependent parts of right and left upper and lower lobes and right middle lobe. Asterisk marks the location of the endotracheal tube

For each human body, visual gradings of dark-field (0–5: none–high) and transmission (0–5: effusion–normal lung) signals of all three readers are shown in ascending order. Colour indicates cases with highest (blue, red) and lowest (light blue, pink) overall median dark-field signal or transmission grading that are shown as individual figures (Figs. 3, 4, 5, and 6)

For transmission radiographs, statistical analysis did not demonstrate significant associations with lung zones (right lung, reader 1, *p* = 0.535, reader 2, *p* = 0.482, reader 3, *p* = 0.312; left lung, reader 1, *p* = 0.568, reader 2, *p* > 0.9999, reader 3, *p* = 0.315).

These results indicate a correlation between the quantity of pulmonary tissue in the beam path (which is lower in lung apex than in middle and lower zones) and dark-field signal magnitude, whereas transmission chest x-rays are indifferent to this aspect.

Dark-field signal and transmission grading in each region of the right and left lung for each reader showed significant correlations only in the right lung lower zone for reader 2 (*p* = 0.0079) and 3 (*p* = 0.0278) x-rays. All other tests did not show significant correlations (reader 1, RL-UZ, *p* > 0.9999, RL-MZ, *p* = 0.437, RL-LZ, *p* = 0.329, LL-UZ, *p* > 0.9999, LL-MZ, *p* = 0.385, LL-LZ, *p* = 0.100; reader 2, RL-UZ, *p* = 0.980, RL-MZ, *p* = 0.929, LL-UZ, *p* > 0.394, LL-MZ, *p* = 0.143, LL-LZ, *p* = 0.077; reader 3, RL-UZ, *p* = 0.827, RL-MZ, *p* = 0.603, LL-UZ, *p* = 0.921, LL-MZ, *p* = 0.185, LL-LZ, *p* = 0.333).

### Intra- and interobserver agreement

The intraobserver agreement was from substantial to almost perfect (*κ* = 0.793–0.971 for dark-field signal and *κ* = 0.790–0.918 for transmission) for visual grading of dark-field and transmission signals (Table 2).

**Table 2:** Intra- and interobserver agreement of dark-field and transmission signal grading

	Reader 1	Reader 2	Reader 3
Intraobserver agreement (*κ* values)			
Dark-field signal	0.959	0.971	0.793
Transmission	0.907	0.918	0.790
Interobserver agreement (*κ* values)			
Dark-field signal			
Reader 1	–	0.848	0.828
Reader 2	0.848	–	0.893
Reader 3	0.828	0.893	–
Transmission			
Reader 1	–	0.772	0.744
Reader 2	0.772	–	0.700
Reader 3	0.744	0.700	–

Visual grading of dark-field signal and transmission shows substantial (weighted Cohen *κ* = 0.61–0.80) to almost perfect (*κ* = 0.81–1.00) intraobserver and interobserver agreement, according to Landis and Koch [29]. Time difference between reading sessions for assessment of intraobserver agreement was 4 weeks

Comparable results were obtained for interobserver agreement (Table 2) that was almost perfect (*κ* = 0.828–0.893) for visual grading of dark-field signal and substantial to almost perfect correlation (*κ* = 0.700–0.772) for visual grading of transmission between all three readers.

### Image quality

Median image quality grading (Fig. 3) assessed by visual evaluation of all nine radiographs and all readers was good (4, interquartile range 1) for left and right lung of dark-field images and very good (5, interquartile range 1) for left and right lung of transmission images. Median image quality grading did not show significant differences between the left and right lung for dark-field (*p* = 0.511) and transmission (*p* = 0.688) radiographs. Median image quality grading was significantly different between dark-field and transmission radiographs for the left (*p* < 0.0001) and right (*p* < 0.0001) lung.

### Correlation of dark-field and transmission radiography with CT findings

**Fig. 7:**
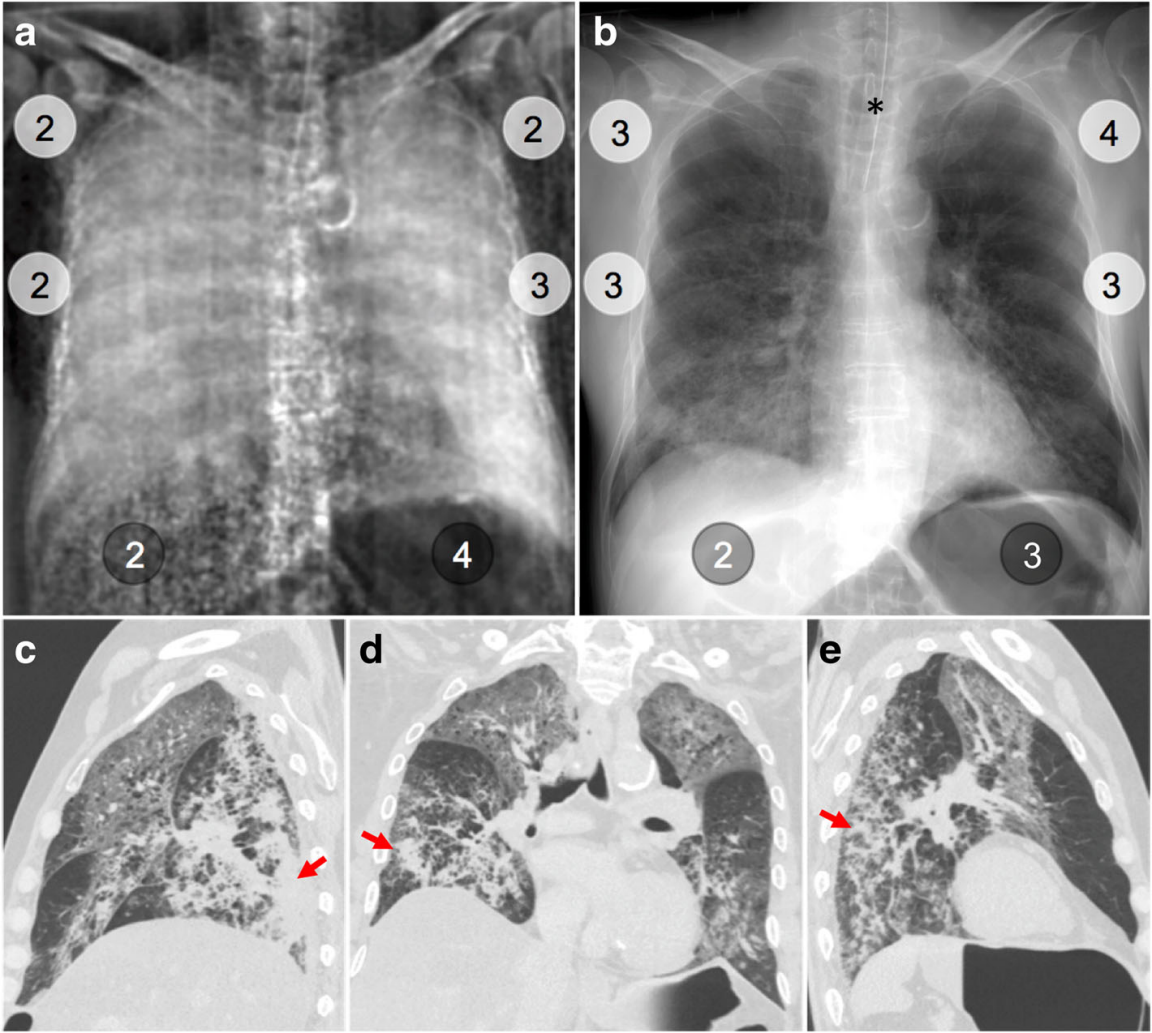
Dark-field chest radiograph, conventional x-ray, and CT of human body 6. Median visual grading (encircled numbers) of x-ray dark-field chest radiograph (**a**) by three independent readers shows markedly reduced dark-field signal in middle and lower zones of the right lung. Median visual grading of transmission in conventional x-ray (**b**) shows reduced transmission in the right lung upper zone as well as right and left lung middle zones and is lowest in right-lung lower zone. Sagittal (**c**, **e**) and coronal (**d**) CT images show interstitial and alveolar infiltrates in the right upper lobe, left upper lobe, and right middle lobe, as well as extensive pulmonary consolidation (red arrows) in the right lower lobe and, to a lesser extent, in left lower lobe. Asterisk marks the location of the endotracheal tube

In the majority of CT images, ground-glass opacities were present in the lungs to a variable extent (Table 3, Figs. 4, 5, 6, and 7). Further pulmonary findings included emphysematous changes, consolidations, tree-in-bud sign, and (interlobular) septal thickening. Extrapulmonary findings were pleural effusions and an enlargement of the heart with haemopericardium.

**Table 3:**
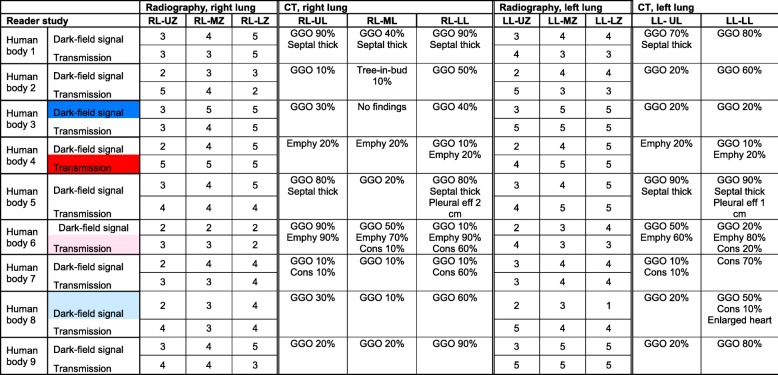
Correlation of dark-field and transmission radiography with CT findings

For all nine human bodies, median dark-field signal and transmission gradings for different lung regions are presented next to computed tomography (CT) findings in pulmonary lobes. Colour indicates cases with highest (blue, red) and lowest (light blue, pink) overall median dark-field signal or transmission grading that are shown as individual figures (Figs. 3, 4, 5, and 6)

*RL* Right lung, *LL* Left lung, *UZ/MZ/LZ* Upper/middle/lower zone, *GGO* Ground-glass opacities, *Emphy* Emphysematous changes; *septal thick* (interlobular) Septal thickening, *pleural eff* Pleural effusion, *Cons* Consolidation, *X%* Percentage of affected lobe

In human body 4 (Fig. 4), we observed only minor (20% of total lobe) emphysematous changes in the parenchyma of all lobes. In the dark-field radiograph, a similar signal increase from the apex to the base of the lung is present in both the left and right lung, whereas transmission radiography shows no differences. The lowest dark-field signal was reported in the lower zone of the left lung in human body 8 (Fig. 5). The corresponding CT revealed moderate ground-glass opacities affecting 50% of the lower lobe and minor (10%) consolidations. Additionally, an enlarged heart with haemopericardium was extending into the left hemithorax. The highest dark-field signal over all zones of the lungs was present in human body 3 (Fig. 6), although a difference between the apex and the middle and lower zones was still visible. In the corresponding CT images, minor to moderate (20–40%) ground-glass opacities were observed in all but the middle lobe. In human body 6 (Fig. 7), the transmission was lowest in the lower zone of the right lung corresponding to widespread (60%) consolidations in the lower lobe and moderate to extensive (50%/90%) ground-glass opacities in the middle or upper lobe, respectively. In addition, extensive emphysematous changes in all lobes were present. Dark-field signal was also low in all zones of the right lung.

## Discussion

X-ray dark-field radiography is a novel imaging modality with high potential for lung imaging that has been translated from an experimental method to clinical applicability in recent years. However, so far, its feasibility for imaging of the human lungs has only been demonstrated in a single post-mortem chest radiograph. Therefore, the purpose of our study was to assess the imaging features of nine post-mortem dark-field chest radiographs from a clinical point of view as a final step before evaluation of dark-field radiography in clinical studies.

In the visual assessment of post-mortem dark-field chest radiographs, we observed a gradient of dark-field signal strength from the apex to the base of the lungs. This can be attributed to an increasing amount of lung parenchyma in the x-ray beam path, as small-angle x-ray scattering increases with the amount of scattering material. However, in conventional chest x-rays, the transmission did not correlate with the dark-field signal, demonstrating that this presents a unique imaging feature of dark-field radiography. This finding is in accordance with results of animal studies [12, 13, 21]. In a clinical context, this would have to be considered when investigating pathologies that decrease dark-field signal. In centrilobular emphysema, a form of chronic obstructive pulmonary disease that primarily affects the upper lobes, knowledge of this finding will be essential for correct image interpretation.

X-ray dark-field radiography is an imaging modality which we believe will primarily be evaluated by visual assessment. Hence, for its clinical application, it is of major importance that imaging findings are consistently reported. We could demonstrate that visual grading of x-ray dark-field signal in the lungs shows substantial to almost perfect intra- and interobserver agreement, comparable to visual assessment of transmission in conventional chest x-rays. These results confirm outcomes of reader studies performed on x-ray dark-field radiographs of different lung pathologies in small animals [12, 14] and underline clinical applicability.

Diagnostic image quality is mandatory for reliable reporting of imaging findings and an insufficient image quality may reduce diagnostic confidence of the reporting radiologist [30]. The image quality of x-ray dark-field and simultaneously acquired conventional radiographs were graded as good and very good, respectively. Therefore, we deduce that x-ray dark-field imaging provides sufficient image quality for its application and evaluation in clinical trials.

Numerous preclinical animal studies have demonstrated the effect of specific lung pathologies on the dark-field signal. For example, in an animal model of idiopathic pulmonary fibrosis [13, 14], x-ray dark-field imaging allowed visualisation of early fibrotic changes in the lungs with dark-field images showing circumscribed areas of markedly reduced dark-field signal in lung parenchyma affected by the pathologic process next to normal areas with high dark-field signal. In our study, the correlation of pathological findings in conventional x-ray and CT images of individual human bodies with dark-field signal intensity showed comparable results in the human lungs. Areas of pulmonary consolidation may contribute to a major reduction of dark-field signal, whereas ground-glass opacities, representing interstitial and alveolar infiltrates, showed an inconsistent effect on the reduction of dark-field signal.

In one human body, an enlargement of the heart correlated with a strong decrease of the dark-field signal in the left lung lower zone, probably by reducing the amount of lung parenchyma in the x-ray beam path. This is a relevant finding as it shows that extrapulmonary pathologies can also influence the dark-field signal intensity and would have to be considered when interpreting clinical dark-field radiographs.

There are several limitations to our study. The application of x-ray dark-field chest radiography to human bodies limited our control over parameters that potentially alter the dark-field signal. Emphysematous changes, interstitial and alveolar infiltrates, pulmonary consolidation, pleural effusion, and an enlargement of the heart are pathologies we observed in the human bodies. Additionally, decomposition processes after death may influence dark-field signal intensity. In this study, it was not possible to differentiate and quantify the individual contribution of a single finding to the change in dark-field signal strength, because none of the lungs was free of pathologies and could have served as a reference. Furthermore, due to lung anatomy, it is difficult to correlate a CT finding in a pulmonary lobe to dark-field signal changes in a lung zone of a radiograph. However, we gained insight into findings that may have a more pronounced effect on dark-field signal reduction, *e.g.*, pulmonary consolidation, that has not been addressed in animal studies. X-ray dark-field imaging was performed in supine position, which leads to dystelectasis in the dorsal basal parts of the lungs and may influence dark-field signal. To at least partially compensate for this, we performed endotracheal intubation and kept airway pressure constant during x-ray dark-field imaging. Since x-ray dark-field radiography is a novel imaging modality, the possibility to train the readers for the visual evaluation of the dark-field images was limited. The number of human bodies included in our study is relatively small. Still, our results are in accordance with previous animal studies and demonstrate clinical applicability.

In conclusion, our study on post-mortem human x-ray dark-field chest radiography demonstrates that x-ray dark-field images provide complementary information of the lungs to conventional x-ray, allow reliable visual quantification of dark-field signal strength, and have reached an image quality warranting an evaluation in clinical trials.

## Additional file

Additional file 1: **Figure S1.** Overview of the dark-field imaging setup, qualitative explanation of dark-field contrast formation, and summary of grating parameters and distances. (DOCX 6811 kb)

## Data Availability

The datasets generated and/or analysed during the current study are available from the corresponding author on reasonable request.

## Abbreviation

CT: Computed tomography
