# Supplementary material for: Imaging features in post-mortem x-ray dark-field chest radiographs and correlation with conventional x-ray and CT
Source: Eur Radiol Exp. 2019 Jul 11;3:25. doi: 10.1186/s41747-019-0104-7 (PMC6620231; doi:10.1186/s41747-019-0104-7)
Supplement: Supplementary file 1 — Figure S1. Overview of the dark-field imaging setup, qualitative explanation of dark-field contrast formation, and summary of grating parameters and distances. (DOCX 6811 kb) [file 41747_2019_104_MOESM1_ESM.docx]

**Additional file 1**


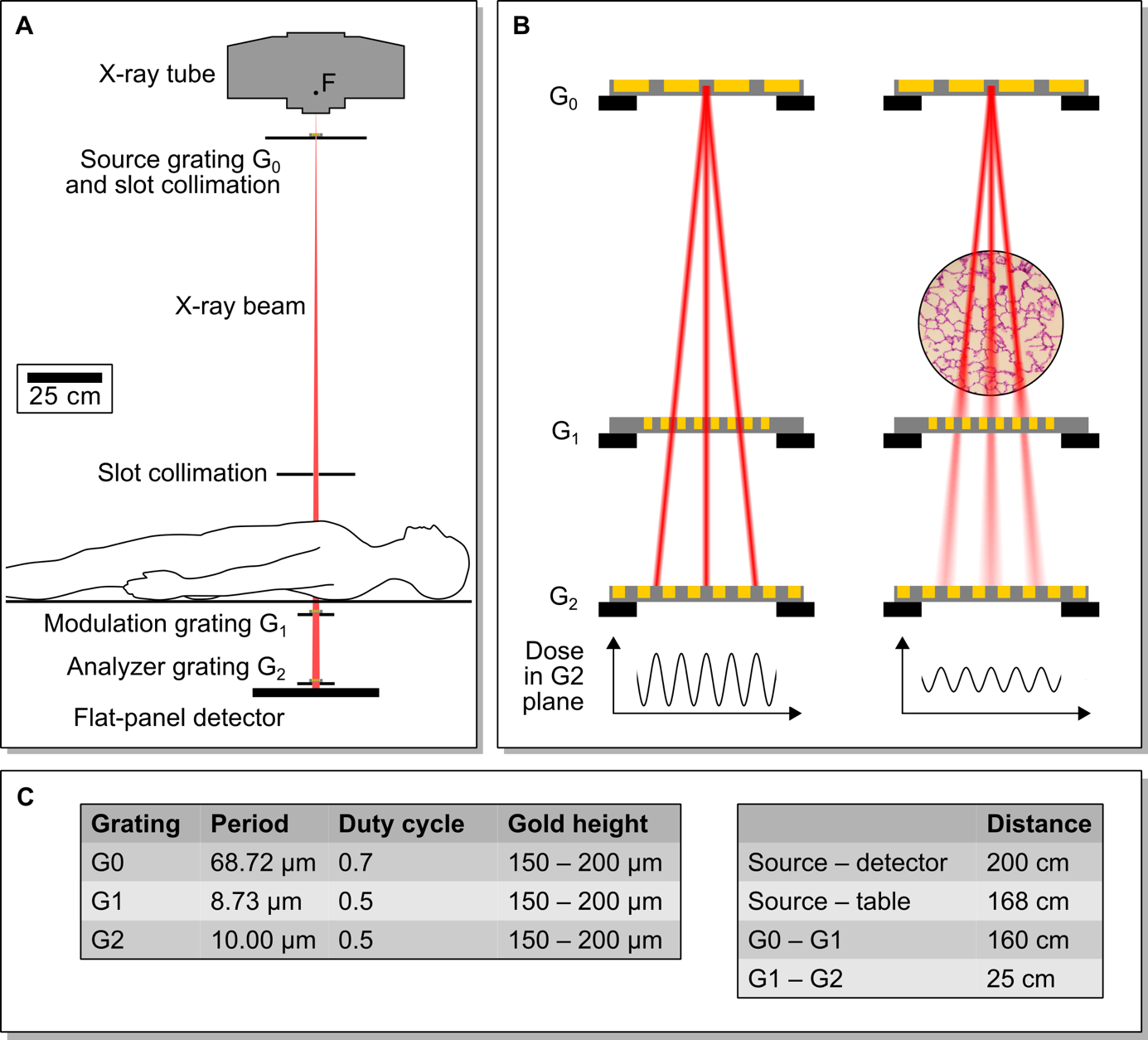


**Figure S1. X-ray dark-field imaging setup, contrast formation and grating parameters.** **(A)** Setup overview, distances approximately to scale. During acquisition, the arrangement consisting of the three gratings and collimators moves in a circular arc around the tube focal spot F, while a large number of frames is recorded. **(B)** Qualitative explanation of the origin of x-ray dark-field contrast. Grating periods and inter-grating distances are selected such that a high-frequency, periodic intensity pattern is generated in the plane of the analyser grating (G_2_). Small-angle scatter due to a sample (*e.g.* lung tissue) leads to a reduction in the relative amplitude (interferometric visibility) of this periodic pattern. The dark-field signal due to a sample is often defined as the factor by which it reduces interferometric visibility. The source grating (G_0_) is necessary to increase the source's transverse coherence length, and the analyser grating (G_2_) converts the high-frequency intensity modulations to lower frequencies detectable with conventional x-ray detectors. **(C)** Salient grating parameters and distances. Photograph of histologic slide: modified from Wikimedia Commons, User "Jpogi", https://commons.wikimedia.org/wiki/File:Alveolar_sac.JPG. Published under CC BY-SA 4.0 (https://creativecommons.org/licenses/by-sa/4.0/).
